# Diabetic concentrations of metformin inhibit platelet-mediated ovarian cancer cell progression

**DOI:** 10.18632/oncotarget.15348

**Published:** 2017-02-15

**Authors:** Rafaela Erices, Sofía Cubillos, Raúl Aravena, Felice Santoro, Monica Marquez, Renan Orellana, Carolina Ramírez, Pamela González, Patricia Fuenzalida, María Loreto Bravo, Bárbara Oliva, Sumie Kato, Carolina Ibañez, Jorge Brañes, Erasmo Bravo, Catalina Alonso, Karen García, Clemente Arab, Vicente A. Torres, Alejandro S. Godoy, Jaime Pereira, Galdo Bustos, Julio Cesar Cardenas, Mauricio A. Cuello, Gareth I. Owen

**Affiliations:** ^1^ Division of Obstetrics and Gynecology, Faculty of Medicine, Pontificia Universidad Católica de Chile, Santiago, Chile; ^2^ Department of Physiological Sciences, Faculty of Biological Sciences, Pontificia Universidad Católica de Chile, Santiago, Chile; ^3^ Universidad Santo Tomás, Santiago, Chile; ^4^ Biomedical Research Consortium of Chile, Santiago, Chile; ^5^ Millennium Institute on Immunology and Immunotherapy, Pontificia Universidad Católica de Chile, Santiago, Chile; ^6^ Hematology and Oncology Department, Faculty of Medicine, Pontificia Universidad Católica de Chile, Santiago, Chile; ^7^ Center UC Investigation in Oncology, Pontificia Universidad Católica de Chile, Santiago, Chile; ^8^ Hospital Gustavo Fricke, Viña de Mar, Santiago, Chile; ^9^ Hospital Sotero del Rio, Santiago, Chile; ^10^ Hospital Luis Tisne, Santiago, Chile; ^11^ Institute for Research in Dental Sciences, Faculty of Dentistry, Universidad de Chile, Santiago, Chile; ^12^ Department of Urology, Roswell Park Cancer Institute, Buffalo, NY, USA; ^13^ Anatomy and Developmental Biology, Institute of Biomedical Science, Geroscience Center for Brain Health and Metabolism, University of Chile, Santiago, Chile; ^14^ Buck Institute for Research on Aging, Novato, CA, USA; ^15^ Universidad Bernardo OHiggins, Facultad de Salud, Departamento de Ciencias Químicas y Biológicas, General Gana, Santiago, Chile; ^16^ Advanced Center for Chronic Diseases (ACCDiS), Faculty of Medicine, Universidad de Chile, Santiago, Chile

**Keywords:** thrombocytosis, hemostasis, EA.hy926, SKOV3, UCI101

## Abstract

Clinical studies have suggested a survival benefit in ovarian cancer patients with type 2 diabetes mellitus taking metformin, however the mechanism by which diabetic concentrations of metformin could deliver this effect is still poorly understood. Platelets not only represent an important reservoir of growth factors and angiogenic regulators, they are also known to participate in the tumor microenvironment implicated in tumor growth and dissemination. Herein, we investigated if diabetic concentrations of metformin could impinge upon the previously reported observation that platelet induces an increase in the tube forming capacity of endothelial cells (angiogenesis) and upon ovarian cancer cell aggressiveness. We demonstrate that metformin inhibits the increase in angiogenesis brought about by platelets in a mechanism that did not alter endothelial cell migration. In ovarian cancer cell lines and primary cultured cancer cells isolated from the ascitic fluid of ovarian cancer patients, we assessed the effect of combinations of platelets and metformin upon angiogenesis, migration, invasion and cancer sphere formation. The enhancement of each of these parameters by platelets was abrogated by the present of metformin in the vast majority of cancer cell cultures tested. Neither metformin nor platelets altered proliferation; however, metformin inhibited the increase in phosphorylation of focal adhesion kinase induced by platelets. We present the first evidence suggesting that concentrations of metformin present in diabetic patients may reduce the actions of platelets upon both endothelial cells and cancer cell survival and dissemination.

## INTRODUCTION

**Figure 1:**
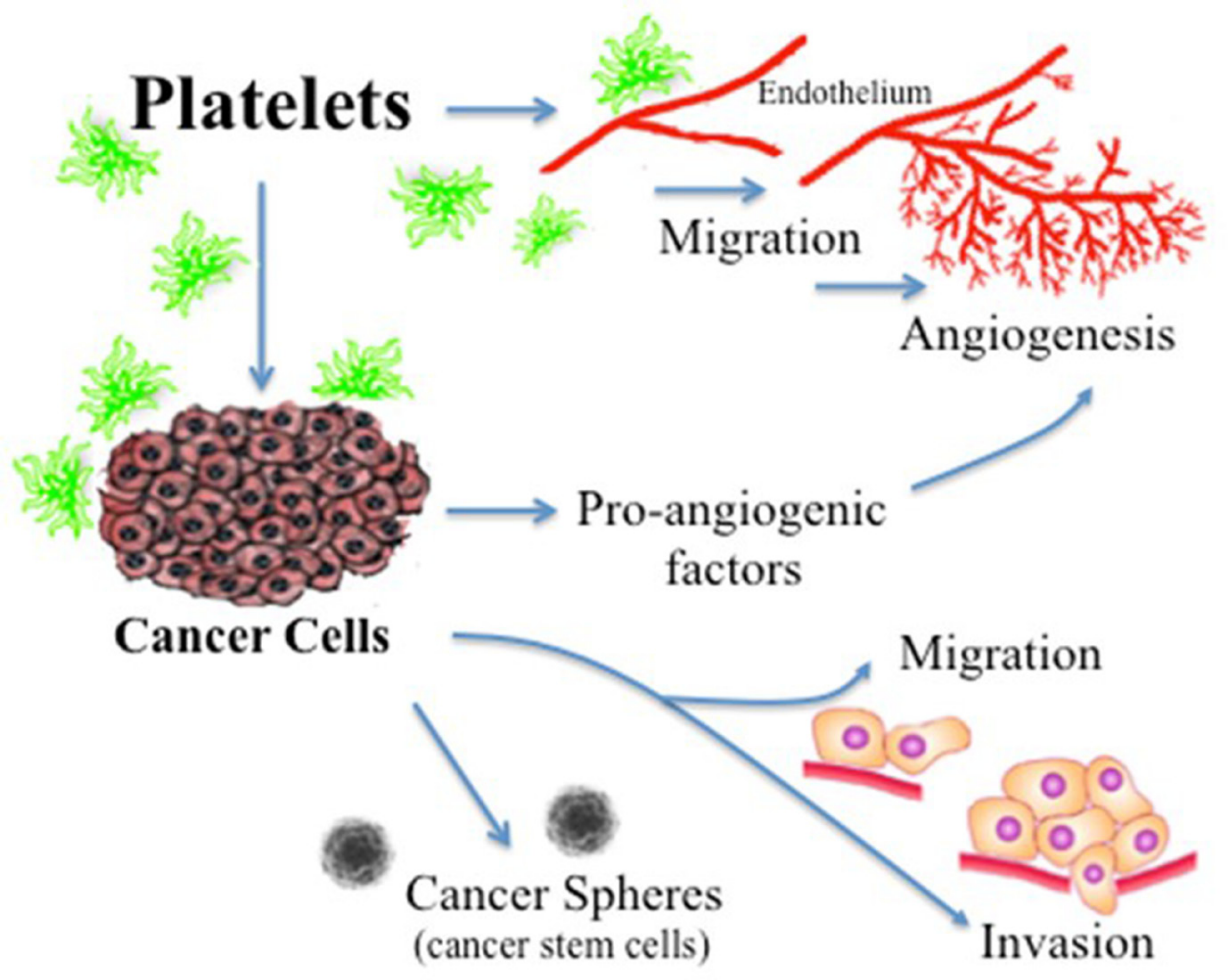
Actions of platelets upon the processes of angiogenesis and tumor promotion Platelets acting directly upon endothelial cells have been reported to enhance the formation of tubular structures and the process of angiogenesis. Platelet acting upon the cancer cell has shown a resultant increase in the liberation of proangiogenic factors, an increase in cancer cell migration and invasion, and an increase in cancer sphere formation.

Ovarian cancer is the fifth leading cause of cancer death in women and the most lethal gynecologic malignancy [1]. Thrombocytosis, high platelets counts, is observed in 10–57% of patients with cancer, which is prevalent in ovarian cancer, and been correlated with poor prognosis [1, 2]. Approximately one-third of women with recently diagnosed ovarian cancer have platelet counts exceeding 450,000/μL [3]. Furthermore, thrombocytosis is associated with several aggressive clinical features including, increased median preoperative serum CA-125 levels, advanced-stage disease and significantly decreased progression-free and overall survival (2.62 years compared with 4.65 years in those without thrombocytosis) [3]. Platelets has a fundamental role in hemostasis and coagulation [4]. Platelets are cytoplasmic fragments lacking a nucleus, which derive from the fragmentation of their precursor cells, megakaryocytes. As summarized in Figure 1, it has been widely reported that activated platelets can act directly upon the endothelium to promote angiogenesis and act upon the cancer cell to contributes to tumor-promoted angiogenesis, tumor-development and metastasis [1, 5]. Platelet activation can occur upon the binding of cell surface agonists such as thrombin [6] or the removal of platelets from their environment, which contains activation inhibitors such as prostaglandins [2, 7]. Platelet activation leads to aggregation, exposure of membrane proteins and release the content of their granules, which in the case of tumor cells is implicated in the promotion of angiogenesis, growth, survival and metastasis [1, 5, 8]. In *in vitro* and *in vivo* murine models it has been shown that platelets participate in cancer development and protect tumor cells in circulation from elimination by the immune system [1]. In a previous work, we showed that platelets could act as chemoattractants to cancer cells, increase the expression of metastasis initiating cell markers and enhance cancer sphere formation (Figure 1) [2]. These influences may enable tumor cells to arrest in the vasculature, mediate an inflammatory response produced by the interaction of platelets with the tumor microenvironment and thus favor proliferation and angiogenesis [1, 9].

Metformin is widely used to treat type 2 diabetes and pre-diabetic syndromes modulating glucose metabolism and fatty acids. Its primary action is to inhibit hepatic glucose production, but it also increases the sensitivity of peripheral tissues to insulin [10]. To date, several epidemiological studies indicate that the use of metformin in patients with cancer would be beneficial, especially observed in an increase in disease-free survival [11, 12]. These studies initiated the scientific interest in determining the mechanism of action by which metformin delivers anti-cancer benefits. Habitual clinical dosing regimens of metformin hydrochloride tablets generally result in steady state plasma concentrations of less than 1 mg/mL, which are achieved within 24 to 48 hours (U.S. Food & Drug Administration). During controlled clinical studies of metformin, maximum plasma metformin levels do not exceed 5 mg/mL (30 μmol/L). Furthermore, it has been reported that the maximum plasmatic concentration in diabetic patients is within a range of 1-4 mg/ml, which corresponds to 6–24 μM respectively (U.S. Food & Drug Administration). Furthermore, Lalau and colleagues showed that the mean + standard deviation plasma concentrations were 2.7 ± 7.3 mg/L (16 ± 44 μmol/L) in a total of 467 patients [13]. However, the concentrations used in most published *in vitro* and *in vivo* studies are several times higher than maximum plasma concentrations that would be achieved with the doses of metformin used by diabetic patients [14]. Thus, the currently published mechanism of action may help promote use of high dose of metformin as a stand-alone cancer treatment, however these mechanisms may not necessarily explain why diabetic concentrations have beneficial effects on cancer incidence and survival [15, 16].

Currently, few studies are available regarding the effects that would have metformin on platelet function. Several studies indicate that in patients with type 2 diabetes mellitus, metformin would be beneficial in maintaining hemostasis in these patients [17]. Several years ago, the effect of platelet function in the presence of metformin was determined, in response to different stimuli, including adrenaline and ADP. Authors observed that the presence of metformin decreases platelet function (aggregation) in response only to the combined stimulus of ADP and adrenaline, but not against simple stimuli [18]. A recent trial [19] has documented that metformin decreased mean platelet volume (MPV), which is known to be increased in diabetes mellitus and has been correlated with vascular complications [20].

Our published results have shown that the use of metformin in concentrations approved for use in diabetics (micromolar range) has no effect on cell proliferation, but can allow ovarian cancer cells to overcome resistance to carboplatin [21]. Furthermore, in a previous publication we demonstrated that platelets could promote cell migration, EMT and sphere formation in cultures of ovarian cancer [2]. Given the increasing literature suggesting that metformin could have beneficial effects upon ovarian cancer patients and that metformin can modify platelet function, we speculate that the use of micromolar concentrations of metformin alone would not have significant effects on angiogenesis, migration and epithelial-mesenchymal transition, but may abrogate platelet-mediated progression of ovarian cancer.

## RESULTS

### Metformin inhibits the increase of endothelial tube formation induced by platelets but does not inhibit platelet-enhanced endothelial cell migration

**Figure 2:**
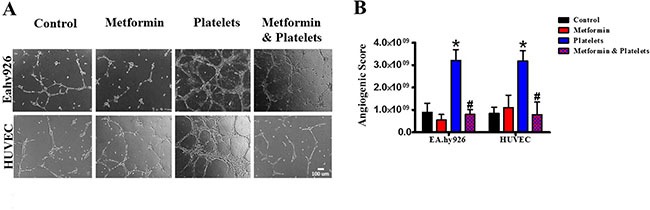
Metformin abrogates the platelet-increased formation of capillary-like structures in matrigel (angiogenesis) (**A**) EA.hy926 cells and HUVEC were seeded onto matrigel and incubated in the presence of control (vehicle), Metformin (20 μM), Platelets (150,000/ μL) and Metformin (20 uM) plus Platelets (150,000/ μL) for 12 hrs. (**B**) Quantified angiogenic score, *n* = 4. *P < 0.05 vs. control, ^#^*P* < 0.05 vs. Platelets. One-way ANOVA followed by Tukey post*-test*.

**Figure 3:**
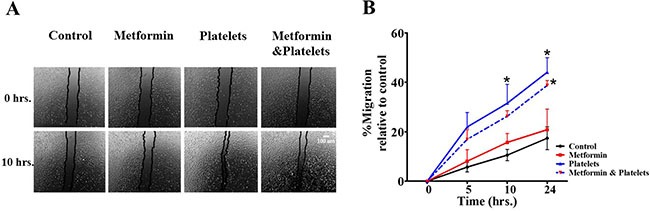
Platelets enhance endothelial cell migration, an effect not inhibited by metformin (**A**) Scratch assay of endothelial cell line EA.hy926 incubated in the presence of control (vehicle), Metformin (20 μM), Platelets (150,000/ μL) and Metformin (20 μM) plus Platelets (150,000/ μL) at 0 and 10 hrs. (**B**) Quantification of the percentage of area respect to the initial scratch area and relative to control (Infinity Analyze) *n* = 3; *P < 0.05 respect to control, two-way ANOVA with Bonferroni post*-test*.

Publications have demonstrated that platelet interaction with the endothelium promotes the process of angiogenesis (upper panel of Figure 1). In this study we utilized platelets at concentrations of 150,000 platelets//μL extracted from healthy volunteers. Although this is a physiological concentration, this quantity of platelets directly in contact with endothelial cells (and further on in this study with cancer cells) within the area of the cell culture dish represents a situation that would only be observed in thrombocytosis. In Figure 2 we confirm that platelets can notably and statistically increase the formation of capillary-like structures in matrigel (hereafter referred to as angiogenesis). We assessed the ability of metformin (20 micromolar) to antagonize this platelet increase in angiogenesis. The co-addition of metformin abrogates this effect, as confirmed in both the endothelial cell line EA.hy926 and in primary cultured Human Umbilical Vein Endothelial Cells (HUVEC). As the process of angiogenesis is known to require endothelial cell migration, we assessed if metformin was inhibiting changes in migration induced by platelets. The scratch assay presented in Figure 3 shows that metformin did not significantly alter the platelet-increased migration of these endothelial cells.

### Inhibition of metformin by compound C reverts its inhibitory effect on platelets-induced-angiogenesis

It is extensively reported that the pleiotropic actions of metformin can be inhibited by compound C (also called dorsomorphin) [22]. This compound which is known to block the AMP-activated protein *kinase* (AMPK) inhibition-independent blockade of Akt/mTOR pathway is also reported to be a selective inhibitor of bone morphogenetic protein (BMP) type I receptors and an activator of the protein kinase A (PKA)-dependent MEK-ERK1/2 signaling pathway [22–26].

**Figure 4:**
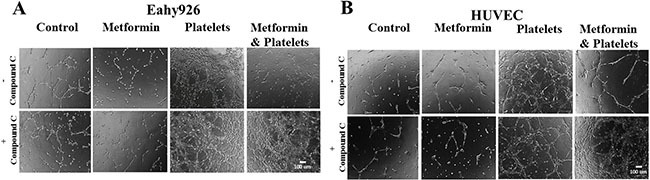
The AMPK inhibitor (Compound C) inhibits the metformin reduction in platelets-increased angiogenesis (**A**) EA.hy926 cells were seeded onto matrigel and incubated with control (vehicles), Metformin (20 μM), Platelets (150,000/ μL) and Metformin (20 μM) plus Platelets (150,000/ μL) for 12 hrs in the absence and presence of Compound C (10 μM). (**B**) HUVEC cells were seeded onto matrigel and incubated with control (vehicles), Metformin (20 μM), Platelets (150,000/ μL) and Metformin (20 μM) plus Platelets (150,000/ μL) for 12 hrs in the absence and presence of Compound C (10 μM).

In the angiogenesis assay, the co-addition of compound C is able to abrogates the inhibitory effect of metformin over the action of platelets, as confirmed in both the endothelial cell line EA.hy926 and in primary cultured Human Umbilical Vein Endothelial Cells (HUVEC) (Figure 4A and 4B, representative images shown from three independent experiments).

In the angiogenesis assay, the co-addition of compound C is able to abrogate the inhibitory effect of metformin over the action of platelets, as confirmed in both the endothelial cell line EA.hy926 and in primary cultured Human Umbilical Vein Endothelial Cells (HUVEC) (Figure 4A and 4B, representative images shown from three independent experiments). Observable differences with compound C were only observed in the platelets-metformin –compound C treatment condition. As previously reported, compound C in our hands inhibited an increase in AMPK phosphorylation induced by micromolar metformin in the SKOV3 cancer cell line, however repeated experiments in the endothelial cell cultures with millimolar metformin failed to show reproducible increases in AMPK phosphorylation or the phosphorylation of its downstream target ACC-1. Thus the mechanism by which compound C inhibits the action of millimolar metformin on endothelial cells is still be elucidated.

### Neither platelets nor metformin alter the cell cycle of endothelial or ovarian cancer cell lines

**Figure 5:**
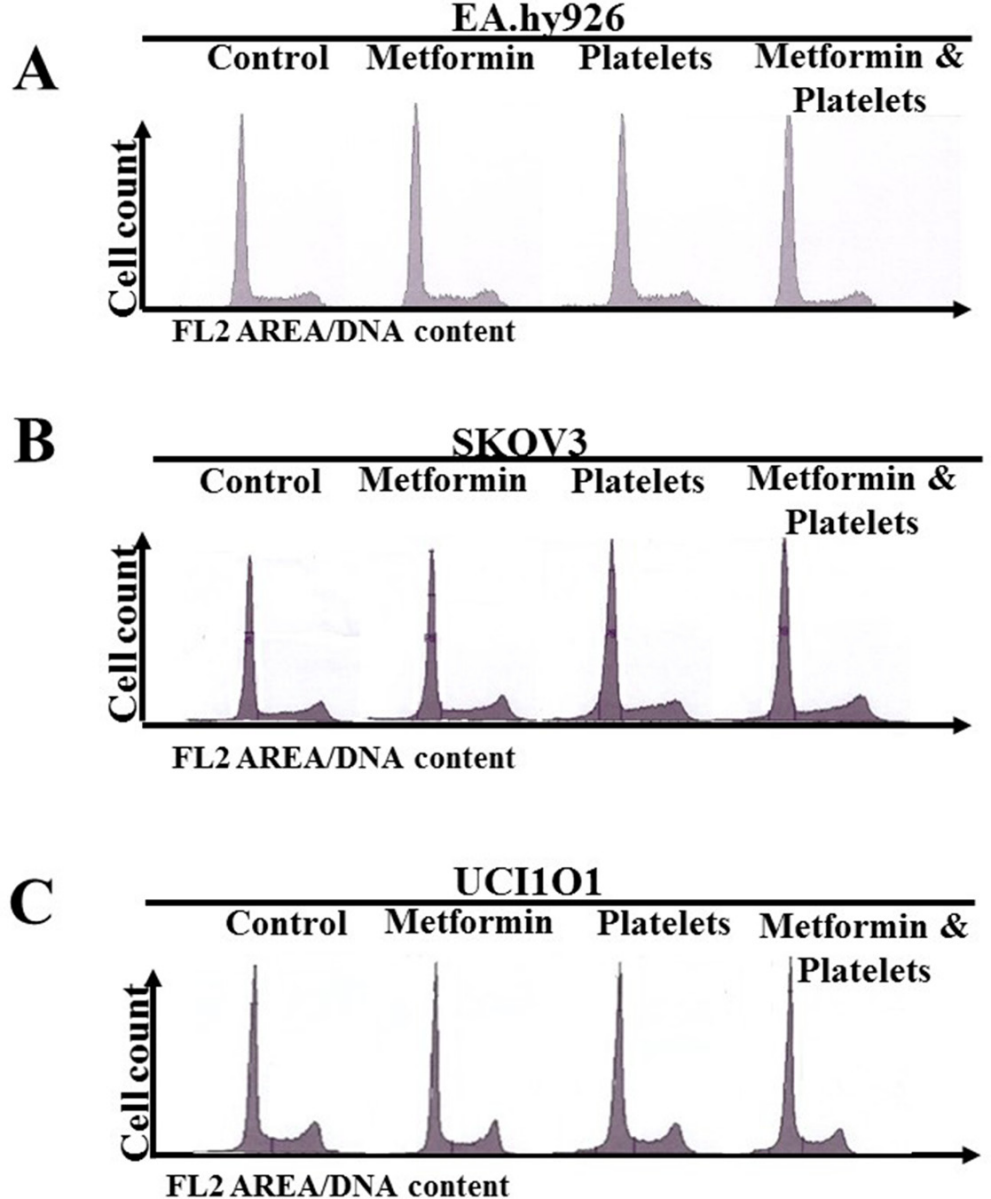
Neither platelets nor metformin alter the cell cycle in ovarian cancer cell lines (**A**) Cell cycle evaluation by flow cytometry of (A) EA.hy926, (**B**) SKOV3 and (**C**) UCI101 incubated in the presence of control (vehicle), Metformin (20 μM), Platelets (150,000/ μL) and Metformin (20 μM) plus Platelets (150,000/μL) at 24 hrs.

It is a possibility that the effects observed by the presence of platelets and metformin could be due to an increase or a decrease in cancer cell proliferation. To address this possibility, EA.hy926 endothelial cells and ovarian cancer cells, were incubated for 24 hours in the presence of platelets or metformin and then proliferation (cell cycle) was assessed by flow cytometry. Figure 5 demonstrates that neither platelets nor metformin alter cell cycle and thus the observed effects are not due to changes in proliferation.

### Platelets enhance the balance of pro-angiogenic factors liberated from ovarian cancer cells, an effect inhibited by metformin

**Figure 6:**
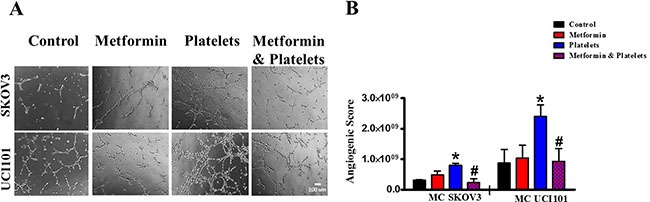
Platelets increase the pro-angiogenic balance of factors liberated from ovarian cancer cell lines, an effect inhibited by metformin (**A**) EA.hy926 cells were seeded onto matrigel and incubated for 12 hrs in the presence of conditioned media of either SKVO3 or UCI101 ovarian cancer cells treated for 24 hrs with vehicle (control), Metformin (20 μM), Platelets (150,000/ μL) and Metformin (20 μM) plus Platelets (150,000/ μL). (**B**) Quantified angiogenic score, *n* = 3, *P < 0.05 vs. control, ^#^ *P* < 0.05 vs. Platelets. One-way ANOVA followed by Tukey post*-test*.

**Figure 7:**
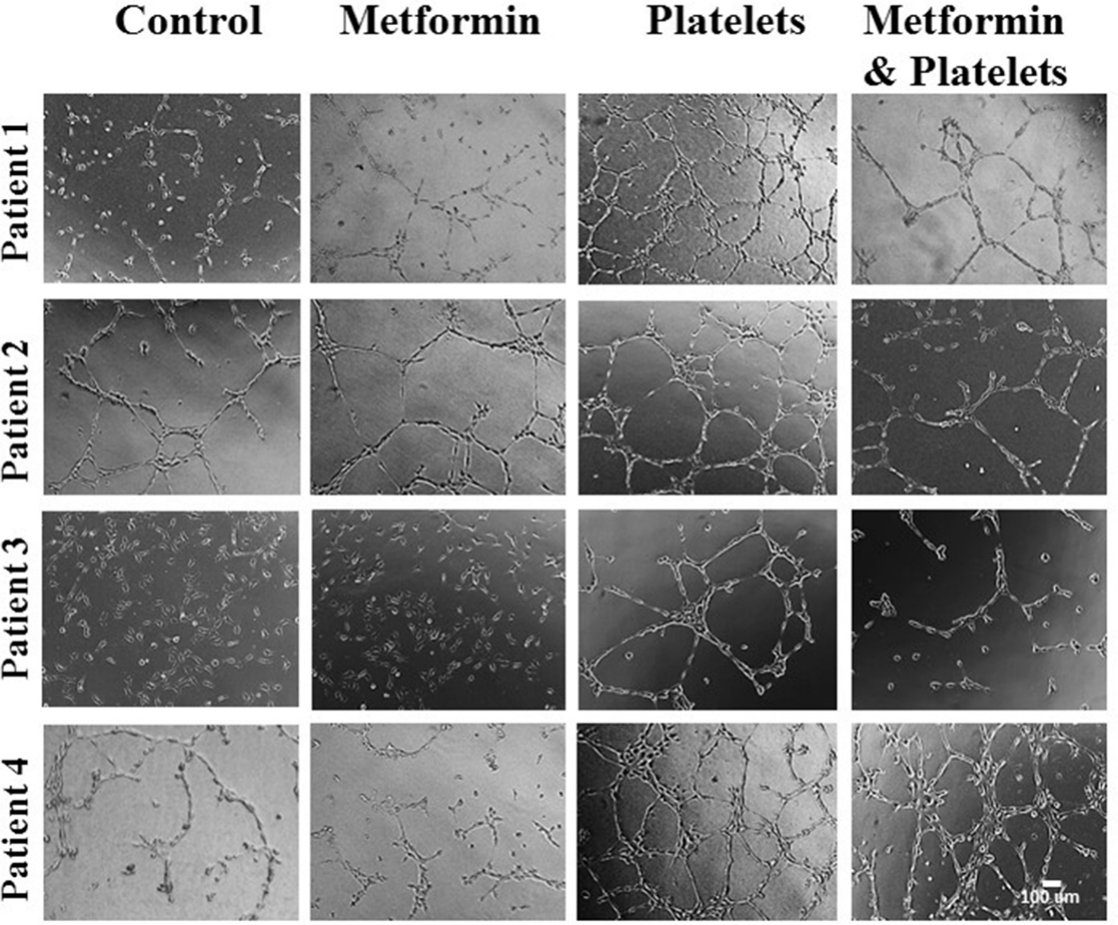
Platelets increase the pro-angiogenic balance of factors liberated from primary cultured ovarian cancer cells, an effect inhibited by metformin EA.hy926 cells were seeded onto matrigel and incubated for 12 hrs in the presence of conditioned media of primary culture from patients treated for 24 hrs with vehicle (control), Metformin (20 μM), Platelets (150,000/μL) and Metformin (20 μM) plus Platelets (150,000/μL). Platelets increased angiogenesis in all patient-derived cultures shown (patients 1–4), with metformin inhibiting this effect in three patient cultures (patients 1–3).

The central focus of this investigation was to determine the role of platelets upon ovarian cancer cells (represented on the lower panels of Figure 1) and whether metformin inhibits this effect. To this end, ovarian cancer cells were co-cultured with platelets in the presence or absence of metformin in the absence of serum. After 24 hours the now conditioned media was removed and used to evaluate (cancer cell promoted) angiogenesis in EA.hy926 cells. As observed in Figure 6A and shown statistically in Figure 6B, platelets increased the balance of pro-angiogenic factors released from both the SKOV3 and UCI101 ovarian cancer cell lines and this was prevented by the presence of metformin. It cannot be determined at the present time is there is an increase in pro-angiogenic factors released by cancer cells or a decrease in anti-angiogenic factors, thus we refer to an increase in the pro-angiogenic balance. A 24-hour incubation of platelets in culture medium in the absence of cancer cells did not significantly increase angiogenesis, demonstrating that the increase in the balance of pro-angiogenic factors originated from the cancer cells (Supplementary Figure 1). We previously published that platelets are activated within seconds after addition to culture medium [2]. To confirm that the metformin inhibition of platelet action was not limited to cancer cell lines, we performed the same experiments in cultures of ovarian cancer cells obtained from the ascites of patients with advanced ovarian cancer. We observed that platelets increased angiogenesis in four of four patient-derived cultures, with metformin inhibiting this effect in three of them. Figure 7 shows representative images of four patient cultures.

### Metformin abrogates the increase in migration, but not the cancer cell invasion, induced by platelets

**Figure 8:**
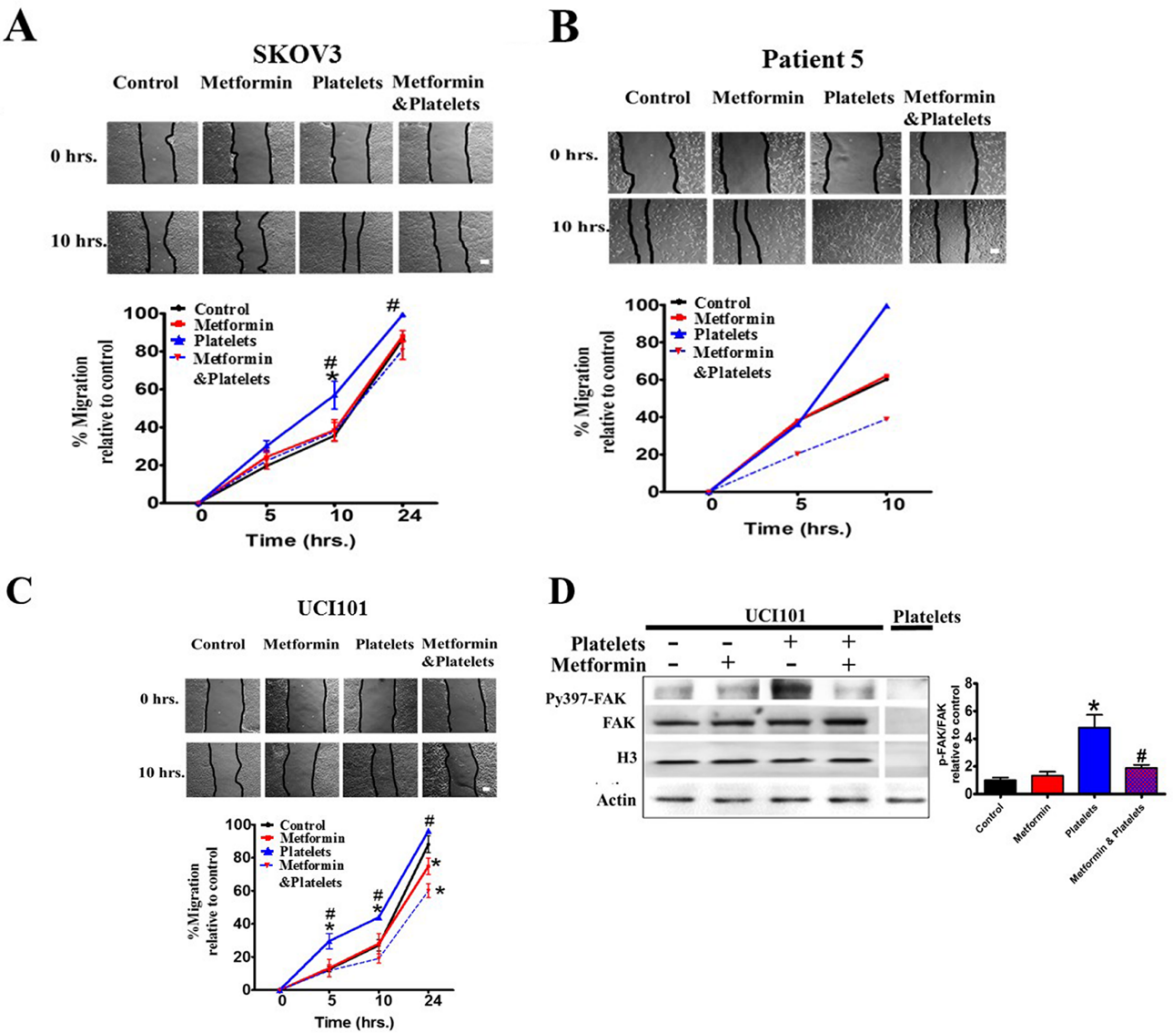
Metformin inhibits cancer cell migration induced by platelets (**A**) Scratch assay of SKOV3, (**B**) primary culture from patient 5 and (**C**) UCI101 respectively, incubated in the presence of control (vehicle), Metformin (20 uM), Platelets (150,000/ μL) and Metformin (20 μM) plus Platelets (150,000/ μL) at 0 and 10 hrs. Quantification of the percentage of area respect to the initial scratch area and relative to control (Infinity Analyze) *n* = 3; *P < 0.05 respect to control, ^#^ *P* < 0.05 vs. Platelets. Two-way ANOVA with Bonferroni as pos*t-test*. (**D**) Western blot analysis of Py397-FAK and FAK total of UCI101 incubated in the presence of control (vehicle), Metformin (20 μM), Platelets (150,000/ μL) and Metformin (20 μM) plus Platelets (150,000/ μL) at 30 min. Densitometry analysis of phospho-FAK/FAK relative to control in UCI101 cells. *P < 0.05 vs. control. One-way ANOVA followed by Tukey post*-test*.

**Figure 9:**
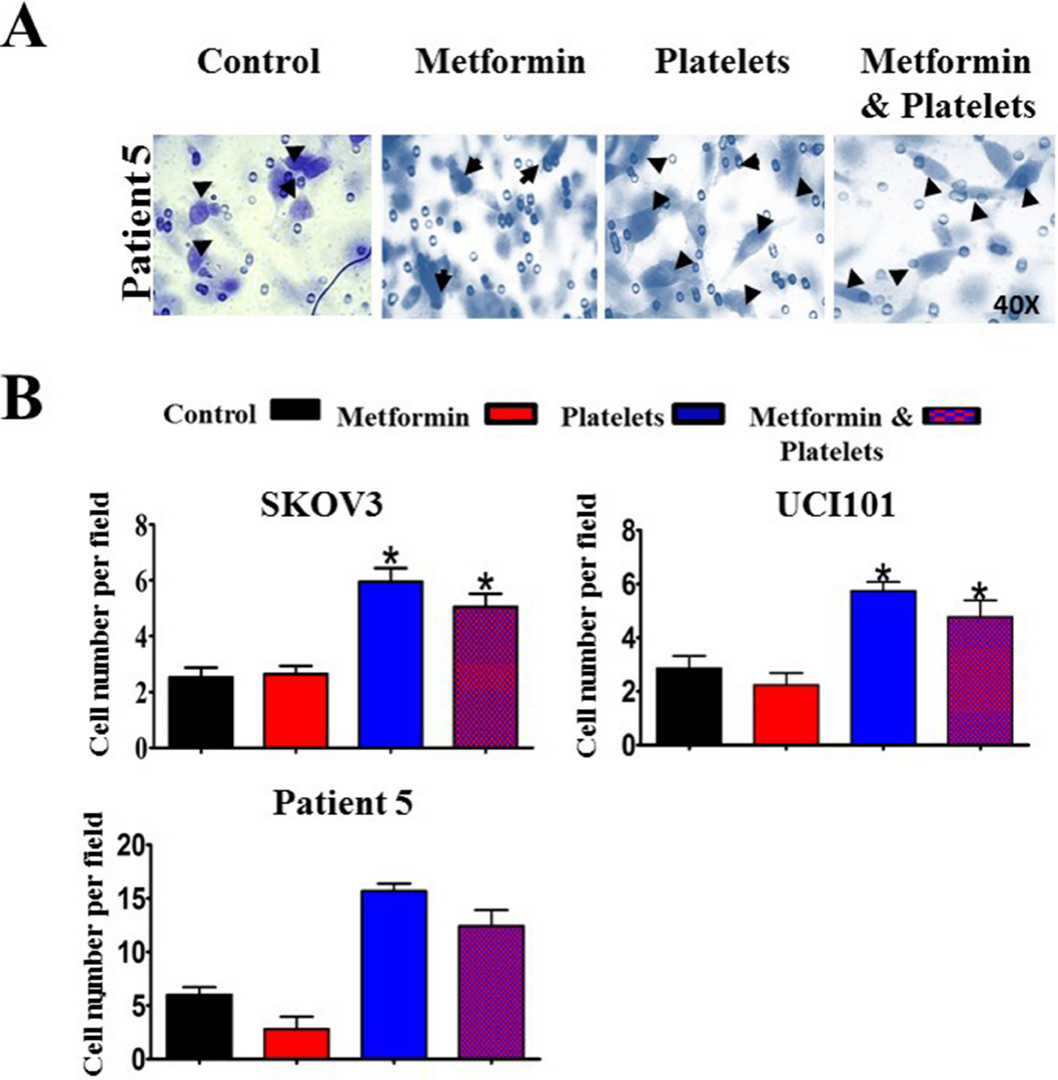
Metformin does not abrogate the increase in cancer cell invasion induced by platelets (**A**) Representative images (40X) of primary cultured ovarian cancer cells (Patient 5) on Matrigel coated Bowden chambers, demonstrating an increase in invading cells (arrows) in the presence of control (vehicle), Metformin (20 uM), Platelets (150,000/ μL) and Metformin (20 μM) plus Platelets (150,000/ μL) (**B**) Quantification of average of cell number per field at 24 hrs in the SKOV3 and UCI101 cells lines and patient 5. *P < 0.05 respect to control, One-way ANOVA with Tukey post*-test*.

Along with a decrease in platelet-induced angiogenesis, we also asked if metformin inhibits other platelet effects, such as cell migration and invasion. Metformin treatment brought about no change in baseline migration, however it significantly inhibited the platelet-increased migration in both cell lines (Figure 8A and 8C) and in a primary culture of ovarian cancer cells (Figure 8B). Further elucidating the mechanism of action we observed that metformin reduced the platelet-increased phosphorylation of the FAK protein, previously reported to be key in cellular migration (Figure 8D). Interestingly, despite a reduction in migration, the presence of metformin did not alter the platelet-mediated increase in cancer cell invasion in both cell lines tested and in a primary cultured ovarian cancer cells (Figure 9A and 9B).

### Platelets increase ovarian cancer sphere formation, an effect inhibited by metformin

**Figure 10:**
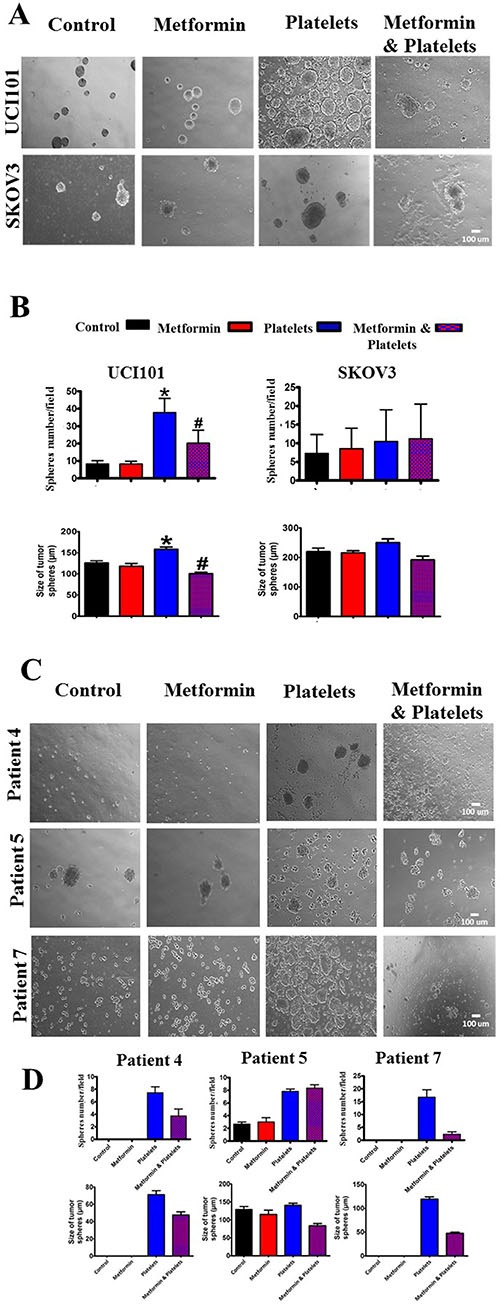
Platelets increase ovarian cancer sphere formation, an effect inhibited by metformin in the UCI101 cell line (**A**) Spheres images (10X) of UCI101 cell line and SKOV3 cell line. (**B**) Quantification of average of sphere number and sphere diameter at day 2. *P < 0.05 respect to control, ^#^*P* < 0.05 respect to Platelets. ANOVA with Tukey post*-test*. (**C**) Spheres images (10X) and quantification of average of sphere number and diameter at day 2 of primary cultures of ovarian cancer cells of patient 3, 4 and 5. (**D**) Standard deviation is shown. Only in Patient 5 did metformin not reduce the increase in sphere formation induced by platelets.

As we recently reported that platelets increase the formation of cell line-derived cancer spheres, we evaluated if this effect was altered in the presence of metformin. In accordance with our previous report [2], platelets increased both the number and average size of cancer spheres formed from the UCI101 cell line, however this was not observed in the SKOV3 ovarian cancer cells (Figure 10A). Metformin inhibited the platelet-mediated increase in both number and size in the UCI101 cell line (Figure 10A). Given this discordance between cell lines we assessed this phenomenon in primary cultures of ovarian cancer cells. As is it shown in Figure 10C and 10D, the culture of cancers cells from three individual patients demonstrated increased sphere formation in the presence of platelets. This effect was notorious in cultures obtained from patient 4 and patient 7 where no sphere formation was observed in the absence of platelets. As observed in the cancer cell line, this increase in sphere formation this was abrogated by the presence of metformin in two of the three patient cultures tested (Figure 10C and 10D).

## DISCUSSION

Metformin is an anti-diabetic drug, widely used for first-line treatment of type 2 diabetes mellitus, that has received a lot of attention from the oncology community as a result of the clinical observation that diabetic patients taking this drug present reduced cancer incidence and increased progression-free survival while undergoing cancer treatment [11, 27]. Despite these clinical observations, the underlying mechanisms of how metformin could be beneficial to cancer patients are poorly defined. Herein, by demonstrating an antagonism of platelet action we shed light on how metformin may reduce endothelial-originated angiogenesis and biological activities associated with cancer progression.

### Platelets and metformin upon the endothelial cell (non-cancerous setting)

In addition to the known effects on glucose metabolism, metformin has been shown to have an anti-inflammatory role in polycystic ovary syndrome, while possessing anti-proliferative actions upon vasculature smooth muscle cells [28]. Metformin has been reported to decrease the release of pro-angiogenic factors in polycystic ovary syndrome, as well as decreasing VEGF levels in obese diabetic patients [29]. Using an air pouch model the authors of the latter mentioned study reported that metformin decreases angiogenesis by 34% in rat granulomatous tissue and further demonstrated *in vitro* that metformin decreases levels of VEGF mRNA and migration of HUVEC cells[30]. However, in our *in vitro* models of an endothelial cell line and primary cultured endothelial cells we observed no effect of micromolar concentrations of metformin on the process of angiogenesis.

The association between platelets and the process of angiogenesis has been reported *in vitro* and *in vivo* [31–33]. Our results confirm the role of platelets in the induction of capillary-like formation of networks in HUVEC cells, and we demonstrate herein that this also occurs in the endothelial EA.hy926 cell line. Additionally, we found that platelets increase endothelial cell migration. We report for the first time that metformin can directly abrogate the *in vitro* angiogenesis induced by platelets, while having no effect on basal or platelet enhanced endothelial cell migration. A possibility is that metformin acts directly upon the platelets to reduces the release of pro-angiogenic factors. It has been reported previously that upon their activation, platelets are capable of releasing and exposing on their surface to both pro-and anti-angiogenic factors depending on the activation stimulus. For example, when platelets are stimulated with ADP they release VEGF, a pro-angiogenic factor of excellence, without releasing endostatin. Upon stimulation with thromboxane A2, platelets can release endostatin in the absence of VEGF, and thus mediate an anti-angiogenic stimulus in HUVEC cells [31]. Although extensive further studies need to be performed, we feel that an exclusive effect of metformin upon the platelets is unlikely as platelet activation occurs when platelets come into contact culture medium (platelets become activated once they are removed from inhibitory factors present in plasma). Herein, we suggest that the inhibitory action of metformin is brought about by the modification of internal signaling pathways in the endothelial cell that respond to platelet stimulation. Metformin is known to signal through the AMPK pathway, however an important observation of this result is that the co-addition of compound C and platelets did not alter the action of platelets, indicating that the action of AMPK is not required for platelet activation or platelet-increased angiogenesis. However, it is still not clear if AMPK phosphorylation is required for the metformin inhibition of platelet action. As mentioned in the results section, in endothelial cell cultures we failed to see reproducible increases in AMPK phosphorylation or that of its downstream target ACC-1 in the presence of micromolar metformin. However, the compound C inhibitor does reproducibly inhibit millimolar metformin action. There are several explanations for this. Firstly AMPK phosphorylation may be occurring but below the levels of detection of the Western blots. However, distinguishing the literature of micromolar metformin from millimolar metformin, there are few publications reporting an increase in AMPK phosphorylation with micromolar metformin and thus the possibility exists that micromolar metformin effects its actions though an alternative pathway [34–37]. While compound C (dorsomorphin) is extensively reported to block the AMPK inhibition-independent blockade of mTOR pathway, it is also reported to be a selective inhibitor of BMP type I receptors ALK2, ALK3 and ALK6 and furthermore an activator of the MEK-ERK1/2 signaling pathway [22–25] Future experiments will determine the exact mechanisms of micromolar metformin action upon the endothelium and the internal signal transduction pathways used by compound C to blunt the response to platelets.

### The platelet-cancer cell interaction and metformin: angiogenesis

The association between platelets and the process of tumor angiogenesis has been recognized [38]. The role of platelets in the regulation of angiogenesis *in vivo* was first documented in a cornea angiogenesis model, in which platelets were shown to support angiogenesis and prevent leakage, and hemorrhage from the newly formed vessels [32]. The participation of platelets in angiogenesis has been observed *in vitro* with the platelets promoting the formation of capillary structures in Matrigel assays [39].

As a first line treatment in diabetes metformin has demonstrated improved peripheral insulin sensitivity and glucose transportation after treatment in diabetic patients [40]. However, other studies have shown that metformin exerts other effects beyond those on glucose metabolism, as by example to modulate inflammation in polycystic ovary syndrome [41] and to exert antiproliferative actions specially in cancer cells [42, 43]. These observations have expanded the range of actions of this drug that may explain several improvements not associated with its anti-hyperglycemic action [28]. Xavier et al., [28] studied the effects of systemic treatment of metformin in the sponge model to evaluate its actions on early steps of the formation of the fribrovascular tissue. They found that metformin has a regulatory function on components of inflammatory angiogenesis, attenuating vascularization.

In the present study, we observed that the addition of platelets to ovarian cancer cells brought about an increase in the balance of pro-angiogenic factors, as demonstrated by the formation of capillary-like structures upon co-incubation of the resulting conditioned medium (removed at 24 hours) with endothelial cells on matrigel. The coincubation of platelets in solely culture medium for the same period before incubation with endothelial cells did not result in changes in angiogenesis, most likely due to the short half-life of the growth factors released by platelets. Interestingly, the presence of metformin reduced this angiogenic potential in both cell lines tested and three of the four cultures of cancer cells isolated from the ascites of high-grade serous papillary ovarian cancer. In one culture, metformin did not reduce platelet-increased angiogenesis. While in the sphere forming assays and the migration assay we clearly see an effect of metformin upon the cancer cell (inhibiting the platelet-effect of the latter two processes) we cannot fully rule out the possibility that Metformin remains in the conditioned medium and thus inhibits angiogenesis by a direct effect upon endothelial cells. However, an anti-angiogenic effect of metformin occurring only upon endothelial cells is unlikely as patient number 4 (Figure 7) showed no effect in the presence of metformin despite the same endothelial cells being used. In other words, if the action of metformin is only upon the endothelial cells, there would be no reason for metformin not to inhibit the platelet-increase in angiogenesis observed in this patient. Possibly, platelets may increase a different milieu of pro-angiogenic factors in each individual cancer and this increase in inhibited or not by metformin. The observation that metformin did not have the same effect on all primary cancer cultures is to be expected as we now know that each cancer is unique, each having specific mutations which alter the responses to external stimuli. Thus, as we have seen throughout oncology treatment, it is difficult to generalize a response to a particular treatment and if metformin is to be used effectively in future cancer treatments, a biomarker to predict response may have to be found. As a further demonstration of the uniqueness of each cancer, as expected the baseline levels of angiogenesis varied for each primary culture (compare controls in Figure 2A), however the presence of platelets increased angiogenesis in each situation.

### The platelet-cancer cell interaction and metformin: migration and invasion

In another assessment of cancer aggressiveness, metformin abrogated platelet-increased migration in both ovarian cancer cell lines and very strikingly in a primary culture (patient 5) despite having no effect on this same process in endothelial cells (please refer to Figure 3A). Despite this inhibition of migration, metformin did not alter the invasion increased by platelets. It is noteworthy that metformin did not alter basal levels of migration and thus cells still have the ability to migrate in the presence of metformin. We can speculate that this basal migratory activity is sufficient for invasion and those platelets increases the proteins (e.g. metalloproteases) involved in degradation of the extracellular matrix via a signaling pathway not altered by metformin.

Interestingly, with respect to migration there is a differing action of metformin upon endothelial cell and the cancer cell. This differing action was further highlighted when we examined the action of the AMPK inhibitor compound C upon the cancer cells. Unlike the endothelial cells, the inhibition of the AMPK pathway abolished the migratory and angiogenic capacities of the cancer cells (data not shown). This may suggest that cancer cells are heavily reliant on the AMPK pathway and possibly its role in autophagy for survival. Whether metformin exerts its effects in cancer cells through AMPK is still the subject of ongoing investigation, however, alternative pathways to the effect of compound C have been reported [44]. However, central to the process of migration in cancer cells is the regulation of focal adhesion kinase (FAK) protein [45]. FAK phosphorylation was observed under basal conditions, however co-incubation of platelets with ovarian cancer cell lines increased significantly FAK phosphorylation and this was inhibited by metformin. In accordance with our findings, an effect of metformin, albeit together with the antibiotic Salinomycin, on FAK has been previously reported in non-small cell lung cancer (NSCLC) cell lines. In NSCLC the combination of both of these compounds reduced the phoshorylation of FAK as well as β-catenin, Src and Chk-2 [46]. In a recent paper it was observed that FAK was present in platelets and was involved in their migration into the tumor [47]. However, in our experiments the levels of FAK in platelets were barely detectable in comparison to levels observed in the tumor cells. Furthermore, the platelets were washed before cancer protein harvesting and thus the increase in FAK phosphorylation observed in Figure 8D corresponds to FAK within the tumor cell. Although results lean towards metformin delivering its anti-platelet action upon the cancer cells, it is still not clear if metformin alters the nature of platelet activation or platelet cancer cell interaction. These extensive experiments are ongoing and will constitute a separate publication, however, hypothesizing that the anti-platelet action occurs through modification of intercellular pathways in the cancer cell, we can speculate that AMP-activated protein kinase (AMPK) is activated by metformin as previously demonstrated [22, 48]. The Unc-51-like kinase 1 (Ulk1) is known to be phosphorylated by AMPK and this protein is reported to form a stable complex with FAK-family interacting protein of 200 kDa (FIP 200) and thus recruiting FAK in its unphosphorylated form [49, 50]. As mentioned, whether metformin via AMPK activation prevents FAK phosphorylation through its incorporation into the Ulk1-FIP200 complex is currently under investigation.

### The platelet-cancer cell interaction and metformin: cancer spheres

Cancer spheres or spheroids are present in the ascitic fluid of ovarian cancer patients, and these are believed to be the key in both chemoresistance and the formation of metastasis [51, 52]. In our previous publications, we demonstrated that platelets are capable of increasing the formation of cancer spheres in ovarian cancer cells [2]. Herein, along with increased sphere number, we further demonstrate that platelets can increase the average diameter of these spheres and showed that their formation was inhibited in the presence of metformin. This may have favorable clinical consequences, as metformin may potentially block both the formation of metastasis initiating cells (MICs) in the primary tumor and the formation of cancer spheres or aggregates of MICs in circulation. However, while an inhibition of sphere formation was observed in the UCI101 cell line and in a patient sample, no such effect occurred in SKOV3, reaffirming that cancer biology remains complex, even in established cell lines. We did not observe any action of metformin alone.

Previous studies with metformin as a single agent reported a reduction in sphere formation in osteosarcoma [43], pancreas [53] and breast cancer derived cells [54]. We feel the difference between these studies and our study lies in the concentration of metformin used. We used micromolar concentrations of metformin that correspond to the levels found in diabetic patients taking this drug, while the other studies used millimolar concentrations. In agreement with our work, in the aforementioned breast cancer study metformin at 10 μM showed no effect, while 500 μM reduced cancer sphere number [54].

## MATERIALS AND METHODS

### Cell line culture

The ovarian cancer cell lines SKOV3 and UCI101 were maintained in Dulbecco's modified Eagle medium (DMEM)/F12 supplemented with 10% fetal bovine serum (Invitrogen, Carlsbad, California, USA). HUVEC were isolated from umbilical cords obtained with patient consents and approved by the ethical committee at the Hospital Clínico Universidad Católica de Chile. HUVEC were obtained by collagenase treatment and maintained in Human Endothelial SFM medium (Life Technologies, Grand Island, NY, USA) supplemented with 10% fetal bovine serum (FBS) and endothelial cell growth supplement (6 mg/ml final concentration) (Merck Millipore, Billerica, MA, USA). These cells were used until passage five. Endothelial EA.hy926 cells were maintained in Iscove's Modified Dulbecco's Media (IMDM) supplemented with 10% fetal bovine serum. For all experiments, endothelial cells were used until passage six. Metformin was solubilized in water (D150959; Sigma-Aldrich, St. Louis, MO, USA), and Compound C (Dorsomorphin) was solubilized in DMSO (ab120843; Abcam, Cambridge, UK).

### Primary cell culture of ovarian cancer

All ovarian cancer samples used in primary cultures were obtained from patients with high-grade ovarian carcinomas, with signed informed consent and with institutional ethical committee approval from the Pontificia Universidad Católica de Chile and all of the participating Chilean hospitals. The hospitals included the Cancer Center at the Pontificia Universidad Católica, Hospital Gustavo Fricke, Hospital Sótero del Río, and Hospital Luis Tisné. For all the experiments, primary cultures were used until passage two.

### Extraction of human platelets

The human platelets extraction was performed as previously described by Orellana et al., 2015 [2]. Briefly, venous blood was collected from healthy volunteers (not taking anti-platelet drugs). Platelet-containing medium was added to the culture dish (final concentration 1.5 × 10^5^ platelets/μL) containing already seeded cell lines SKOV3, UCI101, EA.hy926, HUVEC, and the human ascites primary culture. After 12 hours or 24 hours of incubation, the cell monolayer was washed three times with PBS to eliminate the platelets in suspension.

### Tube formation assay (angiogenesis *in vitro*)

The tube formation assay was performed using EA.hy926 and HUVEC cells as described [55]. Briefly, EA.hy926 cells or HUVEC cells (30,000 cells/well, in DMEM/F12 0% FBS) were plated on top of matrigel coated plates (48 well plates) and incubated with the treatments at 37°C for 12 h. In the case of the treatments of conditioned media from ovarian cancer cell lines SKOV3 and UCI101, these cells were treated for 24 h at 37°C in the presence of either platelets, metformin or both. After 24 h, the conditioned media was removed and centrifuged at 2700 g for 9 min at 4°C to remove cell or platelet debris. This conditioned media was used to resuspend endothelial cells before seeding onto matrigel. Cultures were photographed using 4× and 10× of magnification and all results were quantified using the plugin “Angiogenesis Analyzer” from image J v1.6. For angiogenesis score we used 4X images using the following formula:

Angiogenic score = number of branches × total branch length

### Scratch assay

SKOV3, UCI101 and EA.hy926 cells were cultured in 6 well plates until 100% confluence, and then a vertical and horizontal wound (scratch) was introduced through the cell monolayer using a fine pipette tip. The culture medium was replaced with fresh DMEM/F12 containing 5% charcoal treated serum in the presence or absence of platelets (1.5 × 10^5^ platelets/μL) and or metformin (20 μM). In the case of EA.hy926, the culture medium was replaced with fresh DMEM/F12 in the absence of FBS. Wound closure was assessed by photography at 5, 10 and 24 hours, and quantified using the Infinity Analyze v6.3 (Lumenera Corporation, Ontario, Canada).

### Cell cycle analysis

Flow cytometry (FACSsan, Beckton Dickinson, Franklin Lakes, New Jersey, USA) was performed at Clínica Tabancura, Chile. Briefly, cells were collected and washed 2 times with cold phosphate-buffered saline. The cells were fixed and permeabilized with 70% cold ethanol and incubated with RNase A and propidium iodide (0.5 mg/ml) buffer. Cells pertaining to a sub-G1 fraction, G0/G1 phase, G2/M and S phase were determined as a percentage of the total cell population using the Cell-Quest program (Beckton Dickinson, Franklin Lakes, New Jersey, USA). The sub- G1 fraction was considered as a marker of cell death (apoptosis). Results were gathered from 3 independent experiments.

### Western blot analysis

250,000 cells were seeded in 6 cm plates. Total proteins from cell lines were extracted using 150 mM Tris-HCl lysis buffer containing NaCl 1.5 M, Sodium orthovanadate 0.2 mM and Triton X-100 0.5%. Lysed cells were left for 20 minutes on ice, centrifuged at 7,000 rpm for 7 minutes at 4°C, then cells were sonicated and centrifuged at 7,000 rpm for 7 minutes at 4°C. The resulting protein content of the supernatant was determined by the Bradford method and stored at −80°C. Equal amounts of protein (50 μg/lane) were separated using 12% SDS-PAGE or 10% SDS-PAGE under reducing conditions and transferred to nitrocellulose membranes (Biorad, Hercules, CA, USA), blocked with BSA 5% in TBS-0.1% Tween-20 and incubated overnight at 4°C with the primary antibodies anti-FAK (1:500, rabbit, Cat N° 3285S, Cell Signaling Technology, Boston, MA, USA) and anti-phospho (Tyr397) FAK (1:500, rabbit, Cat N° 3283S, Cell Signaling Technology, Boston, MA, USA). Anti-β-actin (1:3000, rabbit, Cat N° A5060, Sigma Aldrich, St. Louis, MO, USA) and anti-Histone H3 (1:1000, rabbit, Cat N° 9715S, Cell Signaling, Technology, Boston, MA, USA,) both used as load control. All the antibodies were diluted in blocking buffer. The membranes were washed two times for five minutes in TBS-T buffer, incubated with HRP-conjugated anti-rabbit secondary antibody (Biorad, Goat Anti-Rabbit IgG (H + L)-HRP Conjugate; Cat. N°170-6515, USA) for two hours at room temperature and developed with chemiluminescence reagent (Pierce ECL Thermo Scientific, Whaltman, MA, USA). Membranes were exposed to MyECL (Thermo Scientific, Whaltman, MA, USA) and equal protein loading was initially assessed with Ponceau-S red staining (Sigma Aldrich, St. Louis, MO, USA). The integrated optical density of bands was quantitated using the Image J v.1.6 software. The optical densities were expressed as the ratio of treatment/control.

### Invasion assay

Briefly, transwell inserts containing membranes with 8 μm pore size (Nunc) were coated with matrigel (Cat N° 356231, growth factor reduced matrigel matrix, phenol red free, BD Biosciences) as per the manufacturer's instructions. Cells (75,000) were spread over the matrigel (1:5) in 200 μl of medium with no serum, and 800 μl of culture medium with 5% of serum was added to the lower chamber. Cell invasion was studied under the effect of platelets, metformin or both. These treatments were added on the lower chamber of the invasion well. For assessing the number of invaded cells, the filters were fixed with PFA for 30 minutes at room temperature and stained with crystal violet. The filters were mounted on cover slides with Kaiser’s, glycerin-gelatin (Merck). Each experiment was performed in duplicate and was repeated at least 3 times. The inserts were examined using a microscope (Nikon Eclipse E200) with attached camera; photographs of 10 fields at 40X, were obtained per insert, and the number of cells in the underside of the filter was counted. Results were expressed as cell number per field, corresponding to the average of the cells photographed for the correspondent treatment.

### Ovarian cancer sphere formation assay

Ovarian cancer sphere formation assay was performed as previously described by Orellana et al., 2015 [2]. Briefly, 200,000 ovarian cancer cells SKOV3 and UCI101, were cultured with or without 1.5 10^5^ platelets/μL / metformin (20 μM), in 6 well low attachment plates (Nunc^®^, NY, USA) in DMEM/F12 medium supplemented with FGF 50 μg/ml; EGF 0.2 mg/mL; insulin 5 g/ml and 5% of Bovine serum albumin. Images of spheres were taken at 4× and 10× and were counted at day 2, 4 and 6 by microscopy at 10 ×, using 17 fields. The number of spheres was around 2-8 per field. In the case of primary cultures spheres, 15,000 cells were cultured in 12-well low attachment plates (Nunc^®^, NY, USA) in DMEM/F12 medium supplemented with FGF 50 ug/ml; EGF 0.2 mg/ml; insulin 5 g/ml. It should be mentioned that on several occasions, especially after 2 days of culture (data not shown), the co-incubation with platelets of the spheres from cell lines gave rise to large aggregates formed by several spheres trapped within a mesh of platelets. These mesh structures often made quantification difficult and for this reason, quantification was recorded at day 2 of culture.

### Statistical analysis

Experiments were one variable was present were analyzed using ANOVA with the Tukey pos*t-test* (GraphPad Software, La Jolla, CA, USA). When two variables were analyzed a two-way ANOVA with the Bonferroni pos*t-test* was utilized (GraphPad Software, La Jolla, CA, USA). All HUVEC and cell line experiments were performed a minimum of three times. *P* < 0.05 was considered as significant.

## CONCLUSIONS

The published literature is now showing an irrefutable distinction between patient-approved pharmaceutical and super pharmaceutical doses of metformin in the field of oncology. These observations reflect the diversity of action of metformin, further emphasizing that there are still many mechanisms of action upon the cancer cell that need to be identified. This study adds further argument that metformin research should be separated into two disciplines. Firstly, the use of concentrations below 50 micromolar would help elucidate how metformin lowers the cancer incidence and improves outcome in patients taking the drug. Secondly, the use of higher (millimolar) concentrations will evaluate if metformin alone, or when incorporated into future cancer therapeutic regimes, could be beneficial to patient outcome.

We have observed that platelets promote *in vitro* angiogenesis, migration and sphere formation capacity and these processes are inhibited by micromolar metformin, raising the possibility that platelets simply increase cancer cell proliferation, while metformin decreases proliferation. The co-incubation of platelets with ovarian cancer murine cells has been reported to increase cancer cell proliferation [56]. However, in our *in vitro* model we observed no change in proliferation or cell cycle in the presence of platelets. Furthermore, we previously reported that micromolar metformin did not alter the proliferation of SKOV3 or A2780 ovarian cancer cells, which was reaffirmed by Zhang and colleagues [21, 57]. Herein, we confirm that micromolar concentrations of metformin have no effect in SKOV3 cell proliferation and report that this is also true for UCI101 cells.

The results presented in this paper show a protective action of metformin upon platelet-mediation processes involved in cancer progression. This may help us to understand the beneficial clinical effects shown by this drug in patients with ovarian cancer. This anti-platelet action of metformin may not just be limited to cancer. Metformin use has been demonstrated to reduce mean platelet mass and volume in patients at risk for atherosclerotic disease [19]. Thus, in conjunction with positive effects on vascular adhesion, cholesterol levels and inflammatory markers, metformin through the reduction of platelet volume and action may have a role in reducing the development of atherosclerosis. Future investigation will elucidate whether the action of metformin on platelet activity is also relevant in insulin resistance and polycystic ovary syndrome. Herein, we demonstrate that metformin possesses actions that further support the ongoing clinical trials evaluating the inclusion of metformin alongside conventional cancer therapy.

## SUPPLEMENTARY MATERIALS FIGURE



## References

[1] Bambace NM, Holmes CE (2011). The platelet contribution to cancer progression. J Thromb Haemost.

[2] Orellana R, Kato S, Erices R, Bravo ML, Gonzalez P, Oliva B, Cubillos S, Valdivia A, Ibanez C, Branes J, Barriga MI, Bravo E, Alonso C (2015). Platelets enhance tissue factor protein and metastasis initiating cell markers, and act as chemoattractants increasing the migration of ovarian cancer cells. BMC Cancer.

[3] Davis AN, Afshar-Kharghan V, Sood AK (2014). Platelet effects on ovarian cancer. Semin Oncol.

[4] Kuter DJ (1996). The physiology of platelet production. Stem Cells.

[5] Labelle M, Begum S, Hynes RO (2014). Platelets guide the formation of early metastatic niches. Proc Natl Acad Sci USA.

[6] De Candia E (2012). Mechanisms of platelet activation by thrombin: a short history. Thromb Res.

[7] Smith JB (1981). Prostaglandins and platelet aggregation. Acta Med Scand Suppl.

[8] Li N (2016). Platelets in cancer metastasis: To help the "villain" to do evil. Int J Cancer.

[9] Ho-Tin-Noe B, Goerge T, Cifuni SM, Duerschmied D, Wagner DD (2008). Platelet granule secretion continuously prevents intratumor hemorrhage. Cancer Res.

[10] Viollet B, Guigas B, N Sanz Garcia, Leclerc J, Foretz M, Andreelli F (2012). Cellular and molecular mechanisms of metformin: an overview. Clin Sci (Lond).

[11] Romero IL, McCormick A, McEwen KA, Park S, Karrison T, Yamada SD, Pannain S, Lengyel E (2012). Relationship of type II diabetes and metformin use to ovarian cancer progression, survival, and chemosensitivity. Obstet Gynecol.

[12] Kumar S, Meuter A, Thapa P, Langstraat C, Giri S, Chien J, Rattan R, Cliby W, Shridhar V (2013). Metformin intake is associated with better survival in ovarian cancer: a case-control study. Cancer.

[13] Lalau JD, Lemaire-Hurtel AS, Lacroix C (2011). Establishment of a database of metformin plasma concentrations and erythrocyte levels in normal and emergency situations. Clin Drug Investig.

[14] Dowling RJ, Niraula S, Stambolic V, Goodwin PJ (2012). Metformin in cancer: translational challenges. J Mol Endocrinol.

[15] Gritti M, Wurth R, Angelini M, Barbieri F, Peretti M, Pizzi E, Pattarozzi A, Carra E, Sirito R, Daga A, Curmi PM, Mazzanti M, Florio T (2014). Metformin repositioning as antitumoral agent: selective antiproliferative effects in human glioblastoma stem cells, via inhibition of CLIC1-mediated ion current. Oncotarget.

[16] Wan G, Yu X, Chen P, Wang X, Pan D, Wang X, Li L, Cai X, Cao F (2016). Metformin therapy associated with survival benefit in lung cancer patients with diabetes. Oncotarget.

[17] Grant PJ (2003). Beneficial effects of metformin on haemostasis and vascular function in man. Diabetes Metab.

[18] De Caterina R, Marchetti P, Bernini W, Giannarelli R, Giannessi D, Navalesi R (1989). The direct effects of metformin on platelet function in vitro. Eur J Clin Pharmacol.

[19] Dolasik I, Sener SY, Celebi K, Aydin ZM, Korkmaz U, Canturk Z (2013). The effect of metformin on mean platelet volume in diabetic patients. Platelets.

[20] Papazafiropoulou A, Papanas N, Pappas S, Maltezos E, Mikhailidis DP (2015). Effects of oral hypoglycemic agents on platelet function. J Diabetes Complications.

[21] Erices R, Bravo ML, Gonzalez P, Oliva B, Racordon D, Garrido M, Ibanez C, Kato S, Branes J, Pizarro J, Barriga MI, Barra A, Bravo E (2013). Metformin, at concentrations corresponding to the treatment of diabetes, potentiates the cytotoxic effects of carboplatin in cultures of ovarian cancer cells. Reprod Sci.

[22] Zhou G, Myers R, Li Y, Chen Y, Shen X, Fenyk-Melody J, Wu M, Ventre J, Doebber T, Fujii N, Musi N, Hirshman MF, Goodyear LJ (2001). Role of AMP-activated protein kinase in mechanism of metformin action. J Clin Invest.

[23] Huang SW, Wu CY, Wang YT, Kao JK, Lin CC, Chang CC, Mu SW, Chen YY, Chiu HW, Chang CH, Liang SM, Chen YJ, Huang JL (2013). p53 modulates the AMPK inhibitor compound C induced apoptosis in human skin cancer cells. Toxicol Appl Pharmacol.

[24] Kudo TA, Kanetaka H, Mizuno K, Ryu Y, Miyamoto Y, Nunome S, Zhang Y, Kano M, Shimizu Y, Hayashi H (2011). Dorsomorphin stimulates neurite outgrowth in PC12 cells via activation of a protein kinase A-dependent MEK-ERK1/2 signaling pathway. Genes Cells.

[25] Vucicevic L, Misirkic M, Janjetovic K, Vilimanovich U, Sudar E, Isenovic E, Prica M, Harhaji-Trajkovic L, Kravic-Stevovic T, Bumbasirevic V, Trajkovic V (2011). Compound C induces protective autophagy in cancer cells through AMPK inhibition-independent blockade of Akt/mTOR pathway. Autophagy.

[26] Li W, Saud SM, Young MR, Chen G, Hua B (2015). Targeting AMPK for cancer prevention and treatment. Oncotarget.

[27] Evans JM, Donnelly LA, Emslie-Smith AM, Alessi DR, Morris AD (2005). Metformin and reduced risk of cancer in diabetic patients. BMJ.

[28] Xavier DO, Amaral LS, Gomes MA, Rocha MA, Campos PR, Cota BD, Tafuri LS, Paiva AM, Silva JH, Andrade SP, Belo AV (2010). Metformin inhibits inflammatory angiogenesis in a murine sponge model. Biomed Pharmacother.

[29] Jalving M, Gietema JA, Lefrandt JD, de Jong S, Reyners AK, Gans RO, de Vries EG (2010). Metformin: taking away the candy for cancer?. Eur J Cancer.

[30] Soraya H, Esfahanian N, Shakiba Y, Ghazi-Khansari M, Nikbin B, Hafezzadeh H, Maleki Dizaji N, Garjani A (2012). Anti-angiogenic Effects of Metformin, an AMPK Activator, on Human Umbilical Vein Endothelial Cells and on Granulation Tissue in Rat. Iran J Basic Med Sci.

[31] Battinelli EM, Markens BA, Italiano JE (2011). Release of angiogenesis regulatory proteins from platelet alpha granules: modulation of physiologic and pathologic angiogenesis. Blood.

[32] Kisucka J, Butterfield CE, Duda DG, Eichenberger SC, Saffaripour S, Ware J, Ruggeri ZM, Jain RK, Folkman J, Wagner DD (2006). Platelets and platelet adhesion support angiogenesis while preventing excessive hemorrhage. Proc Natl Acad Sci USA.

[33] Packham IM, Watson SP, Bicknell R, In Egginton S (2014). vivo evidence for platelet-induced physiological angiogenesis by a COX driven mechanism. PLoS One.

[34] Cao J, Meng S, Chang E, Beckwith-Fickas K, Xiong L, Cole RN, Radovick S, Wondisford FE, He L (2014). Low concentrations of metformin suppress glucose production in hepatocytes through AMP-activated protein kinase (AMPK). J Biol Chem.

[35] Fasih A, Elbaz HA, Huttemann M, Konski AA, Zielske SP (2014). Radiosensitization of pancreatic cancer cells by metformin through the AMPK pathway. Radiat Res.

[36] Yu H, Bian X, Gu D, He X (2016). Metformin Synergistically Enhances Cisplatin-Induced Cytotoxicity in Esophageal Squamous Cancer Cells under Glucose-Deprivation Conditions. Biomed Res Int.

[37] He L, Wondisford FE (2015). Metformin action: concentrations matter. Cell Metab.

[38] Sabrkhany S, Griffioen AW, Oude Egbrink MG (2011). The role of blood platelets in tumor angiogenesis. Biochim Biophys Acta.

[39] Pipili-Synetos E, Papadimitriou E, Maragoudakis ME (1998). Evidence that platelets promote tube formation by endothelial cells on matrigel. Br J Pharmacol.

[40] Salani B, Del Rio A, Marini C, Sambuceti G, Cordera R, Maggi D (2014). Metformin, cancer and glucose metabolism. Endocr Relat Cancer.

[41] Xu X, Du C, Zheng Q, Peng L, Sun Y (2014). Effect of metformin on serum interleukin-6 levels in polycystic ovary syndrome: a systematic review. BMC Womens Health.

[42] Rattan R, Graham RP, Maguire JL, Giri S, Shridhar V (2011). Metformin suppresses ovarian cancer growth and metastasis with enhancement of cisplatin cytotoxicity *in vivo*. Neoplasia.

[43] Chen X, Hu C, Zhang W, Shen Y, Wang J, Hu F, Yu P (2015). Metformin inhibits the proliferation, metastasis, and cancer stem-like sphere formation in osteosarcoma MG63 cells in vitro. Tumour Biol.

[44] Peyton KJ, Yu Y, Yates B, Shebib AR, Liu XM, Wang H, Durante W (2011). Compound C inhibits vascular smooth muscle cell proliferation and migration in an AMP-activated protein kinase-independent fashion. J Pharmacol Exp Ther.

[45] Sulzmaier FJ, Jean C, Schlaepfer DD (2014). FAK in cancer: mechanistic findings and clinical applications. Nat Rev Cancer.

[46] Xiao Z, Sperl B, Ullrich A, Knyazev P (2014). Metformin and salinomycin as the best combination for the eradication of NSCLC monolayer cells and their alveospheres (cancer stem cells) irrespective of EGFR, KRAS, EML4/ALK and LKB1 status. Oncotarget.

[47] Haemmerle M, Bottsford-Miller J, Pradeep S, Taylor ML, Choi HJ, Hansen JM, Dalton HJ, Stone RL, Cho MS, Nick AM, Nagaraja AS, Gutschner T, Gharpure KM (2016). FAK regulates platelet extravasation and tumor growth after antiangiogenic therapy withdrawal. J Clin Invest.

[48] Cho K, Chung JY, Cho SK, Shin HW, Jang IJ, Park JW, Yu KS, Cho JY (2015). Antihyperglycemic mechanism of metformin occurs via the AMPK/LXRalpha/POMC pathway. Sci Rep.

[49] Loffler AS, Alers S, Dieterle AM, Keppeler H, Franz-Wachtel M, Kundu M, Campbell DG, Wesselborg S, Alessi DR, Stork B (2011). Ulk1-mediated phosphorylation of AMPK constitutes a negative regulatory feedback loop. Autophagy.

[50] Caino MC, Chae YC, Vaira V, Ferrero S, Nosotti M, Martin NM, Weeraratna A, O'Connell M, Jernigan D, Fatatis A, Languino LR, Bosari S, Altieri DC (2013). Metabolic stress regulates cytoskeletal dynamics and metastasis of cancer cells. J Clin Invest.

[51] Soritau O, Tomuleasa CI, Pall E, Virag P, Fischer-Fodor E, Foris V, Barbos O, Tatomir C, Kacso G, Irimie A (2010). Enhanced chemoresistance and tumor sphere formation as a laboratory model for peritoneal micrometastasis in epithelial ovarian cancer. Rom J Morphol Embryol.

[52] Latifi A, Luwor RB, Bilandzic M, Nazaretian S, Stenvers K, Pyman J, Zhu H, Thompson EW, Quinn MA, Findlay JK, Ahmed N (2012). Isolation and characterization of tumor cells from the ascites of ovarian cancer patients: molecular phenotype of chemoresistant ovarian tumors. PLoS One.

[53] Bao B, Wang Z, Ali S, Ahmad A, Azmi AS, Sarkar SH, Banerjee S, Kong D, Li Y, Thakur S, Sarkar FH (2012). Metformin inhibits cell proliferation, migration and invasion by attenuating CSC function mediated by deregulating miRNAs in pancreatic cancer cells. Cancer Prev Res (Phila).

[54] Song CW, Lee H, Dings RP, Williams B, Powers J, Santos TD, Choi BH, Park HJ (2012). Metformin kills and radiosensitizes cancer cells and preferentially kills cancer stem cells. Sci Rep.

[55] Aranda E, Owen GI (2009). A semi-quantitative assay to screen for angiogenic compounds and compounds with angiogenic potential using the EA.hy926 endothelial cell line. Biol Res.

[56] Cho MS, Bottsford-Miller J, Vasquez HG, Stone R, Zand B, Kroll MH, Sood AK, Afshar-Kharghan V (2012). Platelets increase the proliferation of ovarian cancer cells. Blood.

[57] Zhang R, Zhang P, Wang H, Hou D, Li W, Xiao G, Li C (2015). Inhibitory effects of metformin at low concentration on epithelial-mesenchymal transition of CD44(+)CD117(+) ovarian cancer stem cells. Stem Cell Res Ther.

