# Supplementary material for: Diabetic concentrations of metformin inhibit platelet-mediated ovarian cancer cell progression
Source: Oncotarget. 2017 Feb 15;8(13):20865–80. doi: 10.18632/oncotarget.15348 (PMC5400552; doi:10.18632/oncotarget.15348)
Supplement: Supplementary file 1 [file oncotarget-08-20865-s001.pdf]

# Diabetic concentrations of metformin inhibit platelet-mediated ovarian cancer cell progression

## Supplementary Materials

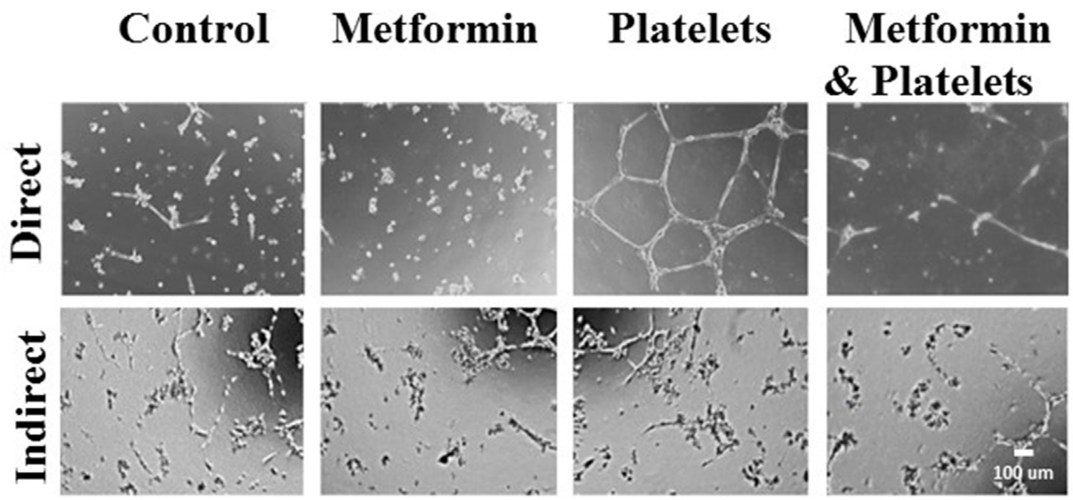


**Supplementary Figure 1: Platelets incubated at 37°C for 24 hours are not capable of increasing the formation of tubular structures in matrigel.** (A) Direct: Endothelial cells (HUVEC) were seeded onto matrigel and incubated in the presence of vehicle (control), metformin (20 uM) platelets (150,000/µL) or metformin and platelets. (B) Indirect: HUVEC were seeded onto matrigel and incubated in the presence of culture media to which vehicle (control), metformin (20 uM) platelets (150,000/µL) or metformin and platelets had been previously added for 24 hrs at 37°C.
